# Pro-Apoptotic Activity and Cell Cycle Arrest of *Caulerpa sertularioides* against SKLU-1 Cancer Cell in 2D and 3D Cultures

**DOI:** 10.3390/molecules28114361

**Published:** 2023-05-26

**Authors:** Rosette Agena, Alejandro De Jesús Cortés-Sánchez, Humberto Hernández-Sánchez, Luis Marat Álvarez-Salas, Oswaldo Pablo Martínez-Rodríguez, Víctor Hugo Rosales García, María Eugenia Jaramillo Flores

**Affiliations:** 1 Ingeniería Bioquímica-Escuela Nacional de Ciencias Biológicas (ENCB)-Instituto Politécnico Nacional, Ciudad de México 07738, Mexico; ragena1900@alumno.ipn.mx (R.A.); hhernan55@hotmail.com (H.H.-S.); ing.oswaldomtz@gmail.com (O.P.M.-R.); 2 Consejo Nacional de Humanidades, Ciencias y Tecnologías (CONAHCYT), UAM-Unidad Lerma, Lerma de Villada 52005, Mexico; aj_cortes@correo.ler.uam.mx; 3 Centro de Investigación y de Estudios Avanzados del Instituto Politécnico Nacional (CINVESTAV), Ciudad de México 07360, Mexico; lalvarez@cinvestav.mx (L.M.Á.-S.); vrosales@cinvestav.mx (V.H.R.G.)

**Keywords:** apoptosis, caspases, cancer, *Caulerpa sertularioides*, cell cycle arrest, lung adenocarcinoma, MMP, SKLU-1 cells, tumor invasion, 2D and 3D culture model

## Abstract

Cancer is a disease with the highest mortality and morbidity rate worldwide. First-line drugs induce several side effects that drastically reduce the quality of life of people with this disease. Finding molecules to prevent it or generate less aggressiveness or no side effects is significant to counteract this problem. Therefore, this work searched for bioactive compounds of marine macroalgae as an alternative treatment. An 80% ethanol extract of dried *Caulerpa sertularioides* (CSE) was analyzed by HPLS-MS to identify the chemical components. CSE was utilized through a comparative 2D versus 3D culture model. Cisplatin (Cis) was used as a standard drug. The effects on cell viability, apoptosis, cell cycle, and tumor invasion were evaluated. The IC_50_ of CSE for the 2D model was 80.28 μg/mL versus 530 μg/mL for the 3D model after 24 h of treatment exposure. These results confirmed that the 3D model is more resistant to treatments and complex than the 2D model. CSE generated a loss of mitochondrial membrane potential, induced apoptosis by extrinsic and intrinsic pathways, upregulated caspases-3 and -7, and significantly decreased tumor invasion of a 3D SKLU-1 lung adenocarcinoma cell line. CSE generates biochemical and morphological changes in the plasma membrane and causes cell cycle arrest at the S and G2/M phases. These findings conclude that *C. sertularioides* is a potential candidate for alternative treatment against lung cancer. This work reinforced the use of complex models for drug screening and suggested using CSE’s primary component, caulerpin, to determine its effect and mechanism of action on SKLU-1 in the future. A multi-approach with molecular and histological analysis and combination with first-line drugs must be included.

## 1. Introduction

Cancer is the second-leading cause of death worldwide after cardiovascular diseases [1]. It is characterized by the uncontrolled and continuous growth of cells that form masses of tumors with the ability to generate metastases. By 2040, it is estimated that the incidence will increase by 47% compared to 2020 [2]. GLOBOCAN reports that lung cancer is the leading cause of death among different types of cancer, with 1,796,144 deaths reported for both sexes, representing 18.2% of cancer-related deaths worldwide and a prevalence of 5.9% over 5 years [3]. Moreover, in 2022, the United States registered 1,918,030 new cancer cases and 609,360 cancer deaths, including approximately 350 deaths per day from lung cancer [4]. Two types of lung cancer are known: small cell lung cancer (SCLC), which represents 15%, and non-small cell lung cancer (NSCLC), characterized by 85% of the incidence of this type of cancer. NSCLC is subdivided into squamous cell carcinoma, large cell carcinoma, and adenocarcinoma. Adenocarcinoma is the most common, representing 40% of lung cancer cases [5].

Treatments to counteract lung cancer include surgery, radiation therapy, chemotherapy, and targeted therapy. However, these generate considerable side effects that decrease the patient’s quality of life, and, despite improvements in diagnosis and treatments in recent years, the prognosis for lung cancer patients is not satisfactory [6]. Natural compounds have a better affinity for interacting with biological systems, need lower development costs, and reduce possible side effects [7,8]. Therefore, alternative treatments with bioactive compounds that can act as adjuvants are sought to reduce the dose of chemotherapeutic drugs. Moreover, the synergism of bioactive compounds with first-line drugs can decrease the drugs’ aggressivity or increase the effectiveness of these drugs while reducing side effects.

Marine organisms, mainly macroalgae, are sources of natural compounds with multiple biological activities, which can decrease the risk of many diseases [9]. The macroalgae *Caulerpa sertularioides* produce a variety of secondary metabolites in response to ecological competition and adverse environmental conditions, among which are alkaloids, carotenoids, phenolic compounds, sterols, pigments, and terpenes, among others [10]. These metabolites have been reported and evidenced as having peculiar chemical structures. In vitro and in vivo study models have found multiple biological activities, including antioxidant, anti-inflammatory, anticancer, antiviral, and antidiabetic activity [11]. Alkaloids, carotenoids, polysaccharides, polyphenols, and terpenoids are biologically relevant due to their multiple anticancer activities [12]. Alkaloids have been attributed to anti-tumor, antiangiogenic, anti-mitotic, and antinociceptive properties [13]. Carotenoids have antioxidant and anticancer activities [14], while phenolic compounds have been attributed to antidiabetic, antioxidant, and anticancer properties [15]. Therefore, *C. sertularioides* is a good candidate for discovering bioactive compounds that will work as adjuvants of pharmacological treatments against cancer. This work aimed to evaluate the cytotoxic, proapoptotic, and anti-invasive activity of the ethanolic extract of *Caulerpa sertularioides* using a three-dimensional model of lung cancer cells SKLU-1.

## 2. Results

### 2.1. Extract Characterization

#### Quantification of Phenols, Flavonoids, Carotenoids, and the Antioxidant Capacity of the 80% Ethanol Extract of *Caulerpa sertularioides* (CSE)

The content of phenolic compounds in macroalgae varies considerably, even in species of the same genus [16]. In this study, the total content of phenols and flavonoids was 81.09 ± 2.28 mg GAE and 70.11 ± 2.06 mg QE/g dry extract, respectively. Carotenoid content was 207.56 ± 2.67 μg Eq. β carotene/g dry extract. The antioxidant capacity of *C. sertularioides* 80% ethanol extract (CSE) evaluated by the ORAC assay was 2171.21 ± 1.35 μmol TE/g dry extract (Table 1).

### 2.2. HPLC-MS Analysis of Extract

#### Identified Compounds of CSE in Negative and Positive Ionization Mode

**Figure 1:**
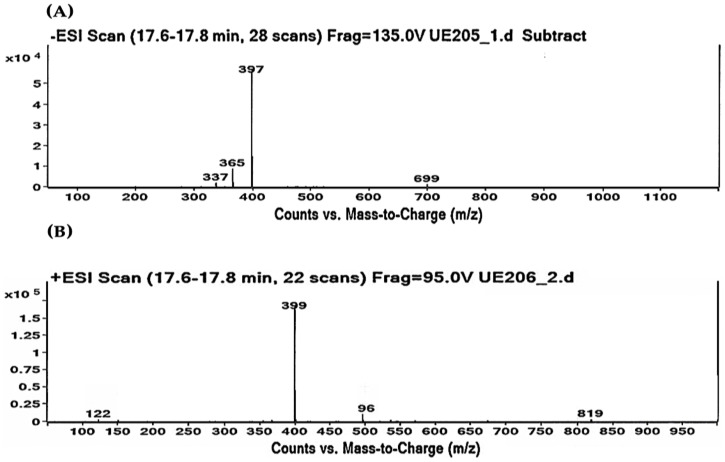
Caulerpin detected by HPLC-MS. (**A**) Negative ionization mode (*m*/*z* = 397). (**B**) Positive ionization mode (*m*/*z* = 399). Retention time: 17.7 min in both polarities.

**Figure 2:**
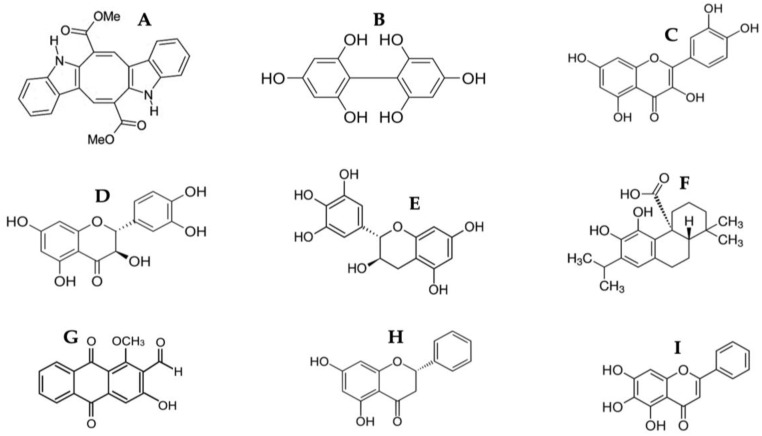
Chemical structure of identified compounds from HPLC-MS of CSE. (**A**) Caulerpin; (**B**) Difucol; (**C**) Quercetin; (**D**) Dihydroquercetin; (**E**) Gallocatechin; (**F**) Carnosic acid; (**G**) Damnacanthal; (**H**) Pinocembrin; (**I**) Baicalein.

As shown in Table 2 and Table 3, the HPLC-MS profile of CSE allowed the tentative identification of phenolic compounds, including phenolic acids, flavonols, flavones, phlorotannins, phenolic diterpenes, and alkaloids. The identification of the phytochemical compounds was established based on the peak area and retention time [17,18]. The main chemical compound identified was caulerpin, which showed ionization in both negative mode (*m*/*z* = 397) and positive mode (*m*/*z* = 399) and presented the same retention time (17.7 min) in both polarities (Table 2 and Table 3). The spectrum mass of the principal compound is shown in Figure 1, and some compounds’ chemical structures are in Figure 2.

### 2.3. Phase 1. Cytotoxic Effects of CSE and Cis in a 2D Culture Model of SKLU-1 Lung Cancer Cell

#### CSE Decreased Cell Viability in a 2D Culture Model

Cell viability is the percentage of healthy cells within a population [19]. Different methods allow the evaluation of cellular health. However, evaluating membrane integrity is the most effective test for detecting dying cells because the latter is crucial for viable cells. In contrast, cells with compromised membranes are considered dead [20].

**Figure 3:**
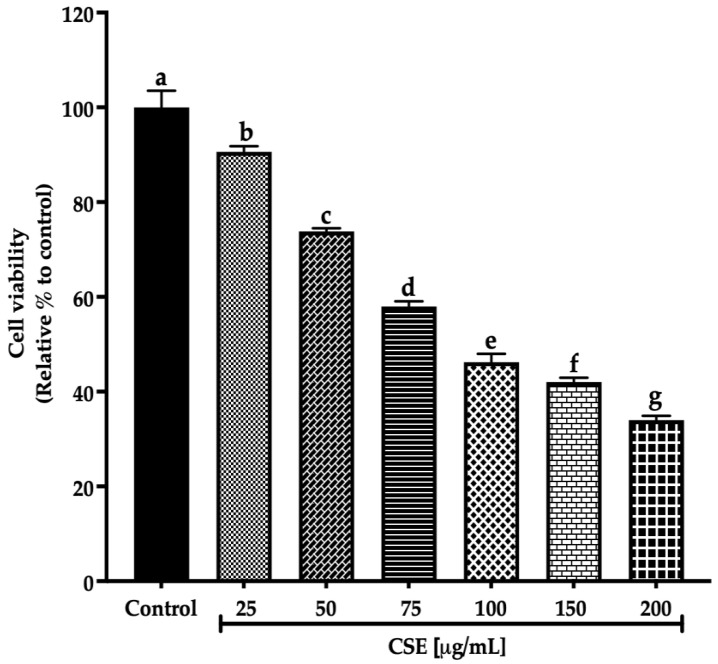
Decreased cell viability in SKLU-1 2D culture model. Cell viability is determined by membrane integrity through the exclusion dye Sytox Green. The 2D model culture was exposed to 25–200 μg/mL of CSE for 24 h. Mean values and standard deviation of percentage of viable cells calculated by Dunnett post hoc test (*p* ≤ 0.05) *n* = 12. Different letters represent significant differences between types of treatment.

This work evaluated the cell viability in a 2D SKLU-1 lung cancer cell culture with the exclusion dye Sytox Green. CSE decreased the cell viability in 2D model culture with an IC_50_ (maximum inhibitory concentration) of 80.28 μg/mL after 24 h treatment exposure (Figure 3).

Ayoub et al. [21] consider a promising IC_50_ for botanicals/crude extract should be up to 100 μg/mL. Therefore, according to these criteria, an IC_50_ of 80.28 μg/mL, registered by CSE after 24 h exposure to SKLU-1, is within this parameter. These findings are the first promising results of the macroalgae *C. sertularioides* on SKLU-1 lung cancer cells.

### 2.4. Phase 2. Cytotoxic Effects of CSE and Cis in a 3D Culture Model of SKLU-1 Lung Cancer Cell

#### CSE Decreased Cell Viability in a 3D Culture Model

**Figure 4:**
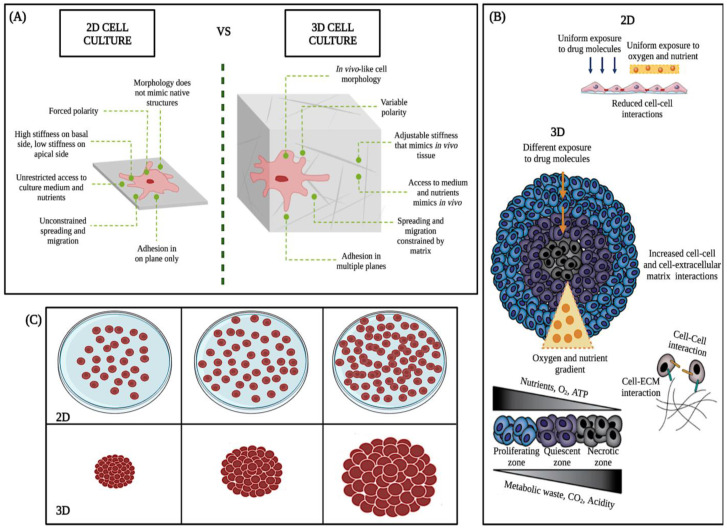
Main differences between 2D and 3D cell cultures. Taken and modified from Kamatar et al. [25]. (**A**–**C**), schematics of the 2D monolayer and 3D cell culture, structural, morphologic, and physiologic differences.

The 3D cell culture model is more resistant and complex than the 2D model (Figure 4A–C). Because of their ability to accurately mimic a tumor’s natural microenvironment, response to stimuli, cell polarity, cell differentiation, protein synthesis, cell morphology, nutrient diffusion gradients, cell-cell and cell-extracellular matrix interaction, and drug metabolism (Figure 4A) [22], in vitro 3D studies have revealed information about tumorigenesis that has not been detectable with traditional 2D models [23,24].

2D culture models allow initial evaluation of the efficacy of anticancer drugs. However, in these experiments, all cells are uniformly exposed to nutrients, oxygen, and drugs, cell differentiation could be better, and the junctions are less accurate than the real junctions (Figure 4B) [26]. In contrast, in vivo, tumors expand as three-dimensional (3D) multicellular masses, where cells have variable and limited access to nutrients, metabolites, and drugs (Figure 4B,C). Therefore, the response of the 3D model to therapeutic interventions is different from that of two-dimensional models (2D) (Figure 4C) [27].

On the other hand, in vivo mouse models are more relevant than the 2D culture model due to complex biology. However, research on living animals raises ethical issues addressed through the 3Rs framework (Replacement, Reduction, Refinement) to reduce animal use [28,29]. Moreover, animal models are expensive, time-consuming, and require resources [30,31]. According to the Food and Drug Administration (FDA) report in 2004, less than 8% of medicinal compounds entering Phase I trials reach the market [25], which might be because mouse models still need to reproduce the complexity of human physiology and metabolism fully. Alternatives to the 2D culture and in vivo mouse models include clinical samples. Still, their limited use due to intratumoral heterogeneity and their many federal regulations [32] makes them routinely low-yielding and challenging to use. Therefore, the 3D model was used to preserve the geometry of typical tumors in vivo.

**Figure 5:**
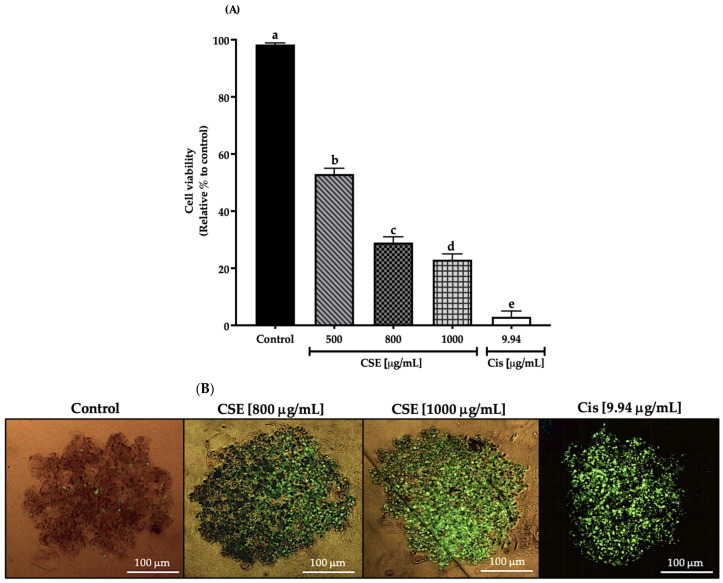
Decreased cell viability in SKLU-1 spheroids (3D). Cell viability is determined by membrane integrity through the exclusion dye Sytox Green. Spheroids were exposed to 500, 800, and 1000 μg/mL of CSE and 9.94 μg/mL of cisplatin for 24 h: (**A**) Decreased cell viability in a 3D model of SKLU-1 cells; (**B**) Images of spheroids taken under an inverted microscope with a scale of 100 μm and an amplification of 20×. Mean values and standard deviation of percentage of viable cells calculated by Dunnett post hoc test (*p* ≤ 0.05) *n* = 12. Different letters represent significant differences between types of treatment.

In this work, cell viability in a 3D SKLU-1 lung cancer cell culture was evaluated with the exclusion dye Sytox Green, registering a decrease in cell viability of 47, 71, and 77% by 500, 800, and 1000 μg/mL of the CSE, respectively, and 97% for 9.94 μg/mL of cisplatin (Cis) (positive control) (Figure 5A). As shown in Figure 5B, cell death is distinguished by the penetration of the dye, which occurs when the cell membrane is compromised, beginning a death process. Considering the results obtained in this experiment, the two concentrations that generated the most significant cytotoxic effect were chosen for the subsequent tests.

### 2.5. Phase 3. Effects of CSE and Cis in a 2D Culture Model of SKLU-1 Lung Cancer Cell

#### 2.5.1. CSE-Induced Apoptosis in a 2D Model of SKLU-1 Cells

In this work, the apoptosis induced by CSE was evaluated by measuring the translocation of Annexin V-FITC and analyzed by flow cytometry.

**Figure 6:**
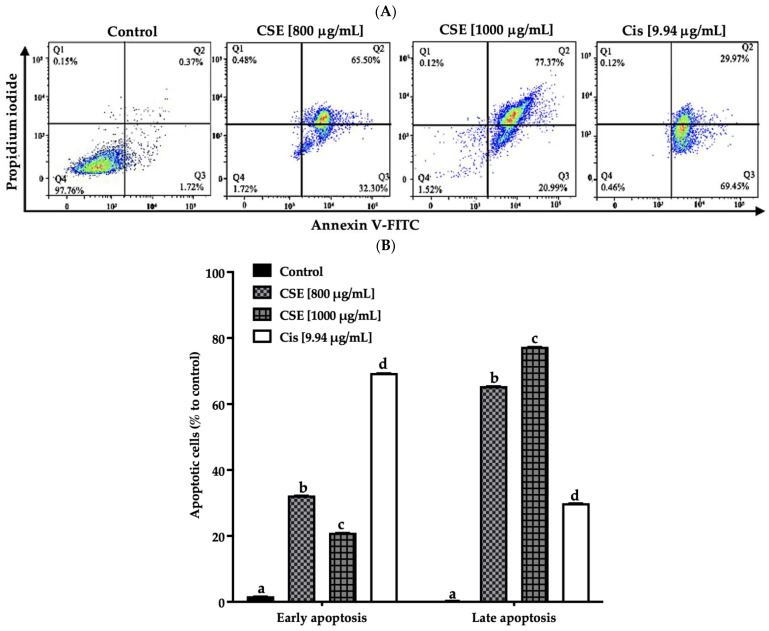
CSE induces apoptosis in SKLU-1 lung cancer. A 2D culture model of SKLU-1 cells was exposed to 800, 1000 μg/mL CSE, and 9.94 μg/mL cisplatin for 24 h. (**A**) Each quadrant indicates the percentage of living cells (annexin V−/PI−), early apoptosis (annexin V+/PI−), late apoptosis (annexin V+/PI+), and necrosis (annexin V−/PI+). (**B**) Total percentage of apoptotic cells. The data represent mean values and standard deviation from independent experiments (*p* ≤ 0.05) *n* = 3. Different letters represent significant differences between types of treatment.

As shown in Figure 6A,B, the exposure of CSE to 800, 1000 μg/mL, and 9.94 μg/mL of cisplatin for 24 h presented a total of 97, 98 and 99% of SKLU-1 apoptotic cells, respectively, compared to the control (<2.5%). Less than 1% of the population, including the control, showed necrotic cells.

#### 2.5.2. CSE Causes Morphological Changes Characteristic of Apoptosis

Multiple biochemical and morphological changes characterize the apoptotic event. The latter is characterized by cell contraction, pyknosis, karyorrhexis, and apoptotic bodies [33]. During cell contraction, the cells become smaller, making the cytoplasm more compact. The chromatin is condensed in pyknosis, and the nucleus often has a slightly irregular outline. Karyorrhexis consists of the fragmentation of the nucleus and the separation of cell fragments into apoptotic bodies consisting of cytoplasm with compacted organelles with or without nuclear fragments [34].

**Figure 7:**
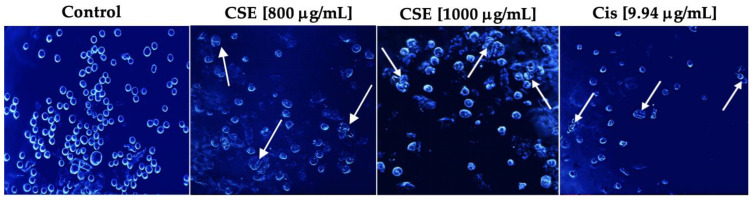
Morphological study of apoptosis in a 2D culture model of SKLU-1 cells. Cells were treated with 800, 1000 μg/mL of CSE, and 9.94 μg/mL of cisplatin for 24h and stained with Hoechst 33258—representative images of morphological changes observed through an inverted fluorescence microscope with 20× amplification. The white arrows indicate the morphological changes generated by the treatments.

As shown in Figure 7, Hoechst stain 33258 indicated that the cells had shrunken, condensed, and fragmented nuclei after exposure to 800 and 1000 μg/mL and 9.94 μg/mL cisplatin for 24 h. The cell morphology of SKLU-1 was severely distorted, and the cells presented very irregular, contracted shapes and showed pyknosis. Unlike the treated cells, the untreated cells emitted a blue fluorescence with an intensity consistent with normal nuclei, a round shape with an intact and healthy membrane, and uniform chromatin. These results indicate that CSE generated morphological changes typical of apoptosis in SKLU-1 cells.

#### 2.5.3. CSE Arrests the Cell Cycle in the S and G2/M Phases in a 2D Culture Model

The duplication of genetic material characterizes the cell cycle and results in two identical daughter cells, each with an exact copy of the genetic material. It consists of two phases: interface and mitosis. This event is negatively regulated by activated checkpoints when DNA damage (ATM/ATR) is detected and by deprivation of growth factors, cytokines, insufficient cell size, lack of molecules for the next phase, and telomere length, among others. The cell cycle checkpoints are critical because the cell can enter the next phase of the cell cycle only through checkpoint testing [35]. The main control points are at the end of the G1 phase (G1/S), in the S phase, at the end of the G2 phase, and at the end of the M phase (M/G1) [36]. Different families of proteins play an essential role in the control points by positively regulating the cell cycle. Of these, cyclins and cyclin-dependent kinases (CDKs) can be mentioned, with the complex cyclin D/CDK 4/6 and cyclin E/CDK2 being the main ones of the G1 phase, cyclin A/CDK2 the main one of S phase, and cyclin A/B/CDK1 the main one of the G2 phase.

**Figure 8:**
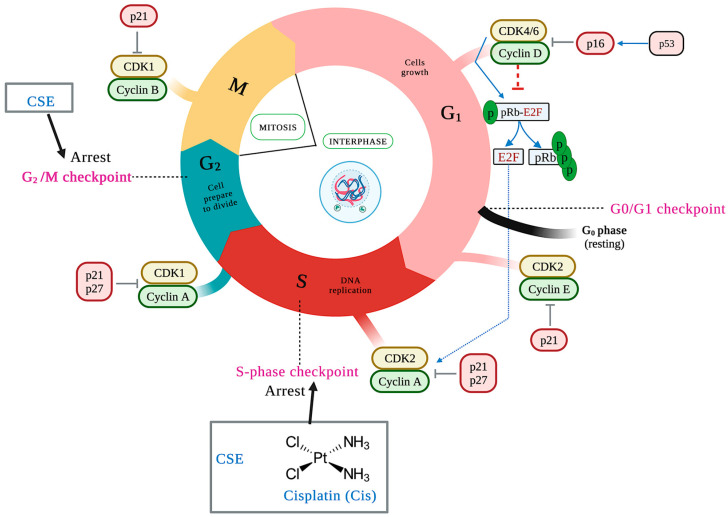
CSE causes cell cycle arrest in the S phase with an accumulation of population in G2/M compared to control. Cells were treated for 24 h and stained with propidium iodide. The black arrows indicate the place of the cell cycle arrest.

On the other hand, there are cyclin-dependent kinase inhibitors (CDKIs), including the INK4 protein family (p14, p15, p16, p18, and p19) that specifically inhibit CDK4 and CDK6 and the CIP/KIP family (p21, p27, and p57) that regulates the cyclin E/CDK2, cyclin A/CDK2, and cyclin B/CDK1 complexes [37]. To determine whether CSE treatment resulted in impaired cell-cycle progression, the cell-cycle patterns of SKLU-1 cells were examined. Compared to the control group, cells treated with 800 and 1000 μg/mL of CSE accumulated in the cell cycle’s S and G2/M phases (Table 4) (Figure 8). The most significant percentage of cells was accumulated in the S phase for cisplatin.

### 2.6. Phase 4. Effects of CSE and Cis in a 3D SKLU-1 Lung Cancer Cell Culture Model on ATP Levels, Membrane Potential Changes, Caspases Activation, and Invasion

#### 2.6.1. CSE Decreased ATP Level in a 3D Culture Model

Adenosine triphosphate (ATP) is the primary energy source for cellular reactions [38] and a critical molecule that maintains and drives the life process by actively participating in metabolic processes. In carcinogenesis, there is a great demand for energy on the part of the cells to guarantee their growth [39].

There are different methods for assessing cell viability. However, the determination based on the quantification of ATP is used the most due to the metabolic dysregulation of tumor cells, one of the hallmarks of cancer [40]. This bioluminescence assay measured ATP, with the light detected directly proportional to the ATP content and indicating the presence of metabolically active cells.

**Figure 9:**
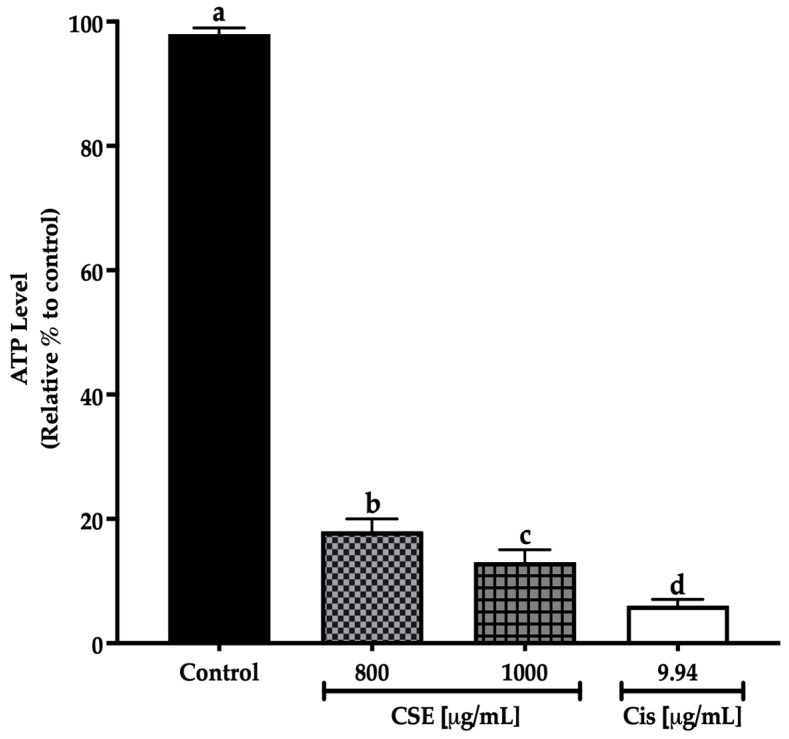
Decreased cell viability in SKLU-1 spheroids (3D model). Cell viability was determined by quantifying the ATP level with the CellTiter-Glo^®^ Reagent 2.0 (PROMEGA) kit. Spheroids were exposed to 800 and 1000 μg/mL of CSE and 9.94 μg/mL of cisplatin for 24 h. Mean values and standard deviation of relative units of ATP luminescence were calculated by the Dunnett post hoc test (*p* ≤ 0.05) *n* = 12. Different letters represent significant differences between types of treatment.

In this work, concentrations of 800 and 1000 μg/mL of CSE showed a significant decrease in the ATP level after 24 h treatment exposure to the control. The decline in the ATP level indicates a reduction in cell viability. The 800 μg/mL concentration decreased the ATP level by 82%; meanwhile, 1000 μg/mL of CSE and 9.94 μg/mL of cisplatin decreased the ATP level by 87 and 93%, respectively (Figure 9). The results in this work show that CSE is a potential therapeutic adjuvant to reduce ATP levels in addition to cell viability in lung cancer spheroids SKLU-1.

#### 2.6.2. Apoptosis Induction

##### CSE Generated a Loss of Mitochondrial Membrane Potential (ΔΨm) in a 3D Model of SKLU-1 Cells

Apoptosis is a regulated process of cell death executed by two alternative pathways: extrinsic and intrinsic. Inducing apoptosis is one of the essential goals of cancer therapy because cancer cells develop abilities to evade cancer mechanisms [41].

The mitochondria are the organelle that generates most of the energy needed for the cell’s biochemical reactions, which is stored in ATP. Mitochondrial membrane potential (MMP) provides information on cellular health. It is essential for ATP production, so a decrease or loss of ATP results in lower ATP production and the release of apoptotic factors that lead to cell death [42].

**Figure 10:**
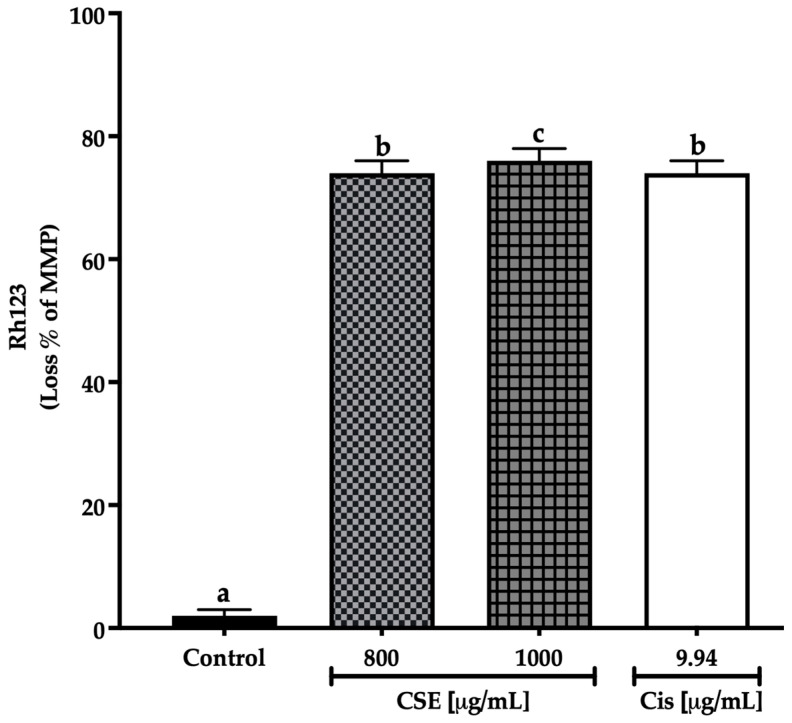
Loss of mitochondrial membrane potential in SKLU-1 spheroids (3D model). MMP determined by Rh123. Spheroids were exposed to 800 and 1000 μg/mL of CSE and 9.94 μg/mL of cisplatin for 24 h. Mean values and standard deviation of relative units of fluorescence by Dunnett post hoc test (*p* ≤ 0.05) *n* = 12. Different letters represent significant differences between types of treatment.

There are different ways to monitor the state of the mitochondria. One of the most frequent is the use of fluorescent dyes that accumulate in healthy mitochondria. This work used rhodamine 123 (Rh123), a cationic dye attracted to the electronegative interior of the mitochondria of viable cells where the fluorescence intensity indicates the mitochondria condition [43]. CSE at 800 μg/mL and cisplatin at 9.94 μg/mL generated an MMP loss of 74%, while CSE at 1000 μg/mL induced a loss of 76% after 24 h of exposure to SKLU-1 spheroids. On the other hand, no change in MMP was observed in the control group. As shown in Figure 10, CSE generated changes in mitochondrial membrane permeability, which correlated with increased cell death and decreased ATP.

##### CSE Induced Apoptosis in a 3D Model by Caspases-3/7, -8, and -9 Activation

Apoptosis is programmed cell death that is executed by extrinsic and intrinsic pathways. This event is mainly triggered by the regulatory activity of caspases, which are enzymes of the cysteine-protease family capable of hydrolyzing tetrapeptides containing an aspartic acid residue [44]. The activation of caspases results in a chain reaction that leads to the activation of other caspases downstream and cell death [45]. Depending on their function, caspases are divided into initiating caspases: -2, -8, -9, and -10, which form the propagation signals of apoptosis, and effector caspases: -3, -6, and -7, which execute this process. Caspase-8 plays a vital role in the extrinsic pathway, while caspase-9 plays an essential role in the intrinsic pathway, where both pathways converge in activating effector caspases [46]. To investigate the pathway and mechanism by which CSE induces apoptosis, the activity of caspases -8 and -9 was evaluated.

**Figure 11:**
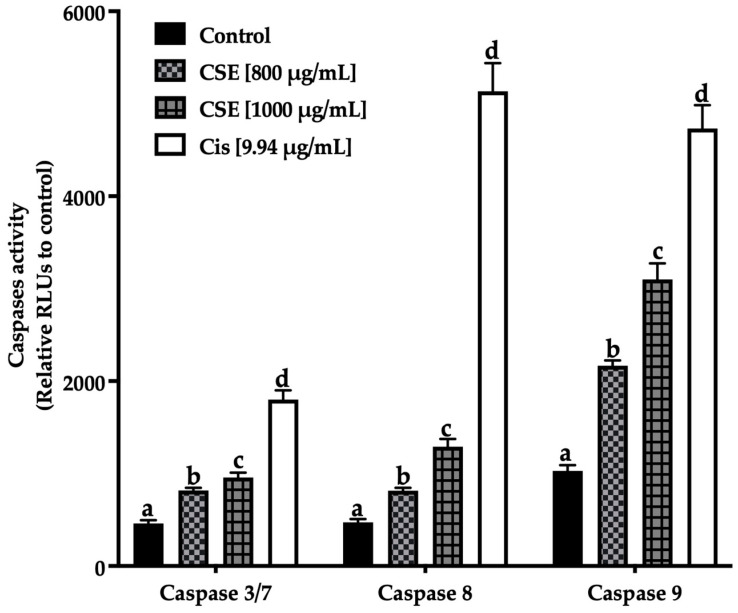
Induction of apoptosis in SKLU-1 spheroids (3D model). Induced apoptosis was determined by caspases-3/7, -8, and -9 activity. Spheroids were exposed to 800 and 1000 μg/mL of CSE and 9.94 μg/mL of cisplatin for 24 h. Mean values and standard deviation of relative units of fluorescence by Dunnett post hoc test (*p* ≤ 0.05) *n* = 12. Different letters represent significant differences between types of treatment.

Figure 11 shows an increase in the initiating caspases (caspases -8 extrinsic pathway, -9 intrinsic pathway) and effectors caspases (-3/7) after being exposed to 800 and 1000 μg/mL of CSE and 9.94 μg/mL of cisplatin. The treatments induced apoptosis in SKLU-1 spheroids after 24 h exposure.

### 2.7. CSE Inhibited Invasion in a 3D Model of SKLU-1 Cells

The invasion process is carried out through three main stages: invasion, intravasation, and extravasation. Invasion is caused by the reduction or loss of intercellular adhesion, allowing the dissociation of an individual or group of cells to the primary tumor mass and by the changes generated in the cell-matrix interaction where the cells acquire abnormally high motility invading the surrounding stroma [47]. This process is characterized by the secretion of substances that degrade the basement membrane (BM) and the extracellular matrix (ECM), showing characteristic markers of epithelial-to-mesenchymal transition (EMT) [48]. Invasion and metastasis are considered hallmarks of cancer because they represent the aggressive nature of cancer [40]. Therefore, inhibiting tumor invasion is critical in discovering drugs and/or therapeutic adjuvants. In this work, cell invasion was determined by the area and perimeter of the invaded matrigel.

**Figure 12:**
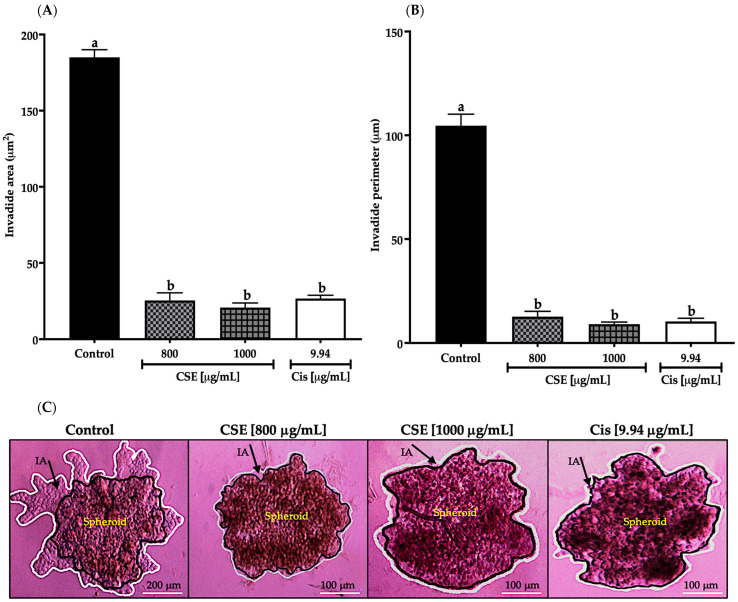
Inhibition of tumor invasion in SKLU-1 spheroids. The area and perimeter of the invaded matrigel determine invasion. Spheroids were exposed to 800 and 1000 μg/mL of CSE and 9.94 μg/mL of cisplatin for 24 h. Mean values and standard deviation of relative units of fluorescence by Dunnett post hoc test (*p* ≤ 0.05) *n* = 6. Different letters represent significant differences between types of treatment. (**A**) Invasion area (μm^2^); (**B**) Invasion perimeter (μm); (**C**) Images of spheroids taken under an inverted microscope with a scale of 100 μm and an amplification of 20×. The black line represents the delimitation of the spheroid, while the white line represents the edge of the invading cells.

As shown in Figure 12A, the invaded area decreased significantly after exposing the spheroids of SKLU-1 cells for 24 h at 800 and 1000 μg/mL; this decrease was even more significant than cisplatin. In Figure 12B, the invaded perimeter shows that CSE concentrations inhibited tumor invasion. The changes that originated in the spheroids due to the cytotoxicity caused by the treatments can be observed in Figure 12C. As shown in Figure 12C, the beginning of the angiogenesis process in the control group can be noticed, confirming the dissociation of the tumor cells from the spheroid, which crossed the BM and ECM gaining new territories. When observing the images of the treated cells, it was concluded that CSE did not allow the detachment of cells from the tumor. These results indicated that CSE could directly inhibit the invasive potential of SKLU-1 cells.

## 3. Discussion

The total phenols content obtained from the algae *C. sertularioides* in this work was higher than those reported with a methanolic extract of *Caulerpa racemosa* and *Caulerpa lentillifera* (10.33 ± 0.02 and 4.52 ± 0.42 mg GAE/g dry extract, respectively) [49]. A similar phenol content was reported using a methanol extract from the algae *C. racemosa* (19.8 ± 2.01 mg GAE/g dry extract) [50]. Values such as 73 ± 2.08 mg GAE/g dry extract were reported using an ethanol extract from the algae *C. lentillifera* [51]. The flavonoids obtained in this study were between 2.85 and 14.22 times higher than that reported by [49] in the species *C. racemosa* and *C. lentillifera* (24.52 ± 2.17 and 4.93 ± 0.27 mg QE/g of dry extract, respectively) using a methanol extract. Previous studies have found significant variations in the chemical components of the same species and/or genus due to seasonal changes, geographical and environmental conditions, extraction method used, and solvent used, among others [52].

Referring to carotenoids, Balasubramaniam et al. [53] reported a lower carotenoid content (195 ± 0.00 μg β carotene/g dry extract) compared to that obtained in this study (207.56 ± 2.67 μg Eq. β carotene/g dry extract). However, these researchers used the species *C. lentillifera* as raw material. These variations in the content of β carotene and other pigments in some macroalgae species vary considerably, as these are affected temporally and spatially [54].

Antioxidants are essential at nutritional and physiological levels because they prevent, protect, delay, and/or eliminate oxidative damage caused by reactive oxygen species (ROS) to cell membranes, mitochondria, DNA, lipids, or proteins during aerobic cell metabolism [55]. The antioxidant capacity evaluated by the ORAC assay in this work showed that *C. sertularioides* 80% ethanol extract (CSE) (2171.21 ± 1.35 μmol TE/g dry extract) has greater antioxidant capacity against peroxyl radicals than an ethanolic extract of *C. racemosa* (683.72 ± 15.86 μmol TE/g of dry extract) [53]. Likewise, two aqueous extracts of the algae *Fucus vesiculosus* had an antioxidant capacity of 1540 ± 220 and 1840 ± 3 μmol TE/g of the dry extract [56]. The antioxidant capacity of the ethanolic extract is significant because one of the mechanisms that reduce cell viability and invasion is fighting the oxidative stress generated in the cellular environment, which is one of the common imbalances of chronic diseases. Different studies have shown a close relationship between the content of phenolic compounds and the high antioxidant capacity of a substance or food, which is no exception for macroalgae because these compounds have been considered one of their most effective antioxidants [52]. Carotenoids have also been recognized for their anti-oxidative properties due to their ability to eliminate and deactivate free radicals [57]. Therefore, the high antioxidant capacity of *C. sertularioides* is attributed to the high content of phenols, flavonoids, and carotenoids.

CSE decreased the cell viability in a 2D culture model with an IC_50_ of 80.28 μg/mL after 24 h treatment exposure. The HPLC-MS analysis revealed the presence of caulerpin (CLP), an alkaloid, in 80% of the algae species of the *Caulerpa* genus; however, it can be found in other algae genera [58]. Various biological activities have been attributed to it, including anticancer, antiviral, and antimalarial activities [50]. Table 5 shows the cytotoxic effect of caulerpin (CLP), the main compound that was identified in CSE by HPLC-MS but isolated and purified from other seaweed species on different cancer cell lines in a 2D culture model with relatively low IC_50_ (from 7.97 to 47.4 μg/mL in a dose-dependent manner) [59,60,61,62,63]. These variations in the IC_50_ might depend on the cancer cell line, the initial cell seeding density, and CLP’s nature, source, and extraction method. According to He et al. [64], IC_50_ errors due to differences in the proliferation rates and enzyme activity of cancer cells were not mentioned in any articles. The authors reported that only 27.6% (8/29) of the manuscripts said per-well seeding numbers (i.e., cell densities), and the other papers did not provide such information. Carnosic acid was detected, the main phenolic diterpene of the Rosmarinus officinalis L. plant (commonly known as rosemary). In a dose-dependent manner, carnosic acid inhibited the cell viability of three colorectal cancer cell lines from a two-dimensional model: Caco-2, HT29, and LoVo, with IC_50_ values from 7.98 to 31.91 μg/mL [65]. Likewise, the presence of dihydroxyflavanone and pinocembrin is related to the decrease in cell viability, proliferation, and autophagy in lung carcinoma cells, A549, in a dose-dependent manner.

In this work, the IC_50_ in the 2D and 3D culture models was 80.28 and 530 μg/mL, respectively, after 24 h of CSE exposure on SKLU-1 cells for both models. The IC_50_ obtained in the 3D model is understandable because cells in 3D models (spheroids) are more resistant to pharmacological treatments. Table 6 shows different reports on lung carcinoma cells (H460, A549, and H1650), showing that the IC_50_ of the same drug on the same cell line increases significantly in the 3D model compared to the 2D model [66]. Although the IC_50_ of these drugs (Table 6) is very low compared to that of CSE, they cause significant side effects such as anemia (decrease in erythrocytes), leukopenia (decrease in leukocytes), neutropenia (reduction of neutrophils), thrombocytopenia (decrease in platelets), nausea, and vomiting, among others, causing the patient to be more vulnerable to other diseases and resulting in a lower quality of life. The IC_50_ fluctuates between 10.39 and 66.51 times higher when comparing both models (Table 6) while, in this work, the difference is less than ten times.

In an additional study, the same concentration of fucosterol and its combination with 5-fluorouracil, which causes cytotoxicity in a 2D colon carcinoma model (HCT116 and HT29), was ineffective in the three-dimensional model [67]. Likewise, Malhão et al. [68] did not detect cytotoxic and antiproliferative activity in the three-dimensional breast carcinoma model (MDA-MB-231) after exposing the cells to 2.06 μg/mL of fucosterol for 96 h. In the same way, a concentration of 6.59 μg/mL of fucoxanthin did not present a cytotoxic effect on the spheroids of MDA-MB-231 cells until its combination with 0.54 μg/mL of doxorubicin, decreasing cell viability by 22% through the MTT assay. Similarly, cytotoxicity was generated on the spheroids MDA-MB-231 through the LDH assay because of combining 13.18 μg/mL of fucoxanthin with 1.09 and 2.72 μg/mL of doxorubicin, releasing LDH at 56 and 77 %, respectively [69].

The translocation of phosphatidylserine (PS) from the cytoplasm to the outer layer of the plasma membrane is one of the most relevant alterations that occur on the cell surface under the induction of apoptosis. Annexin V is a 35 kDa protein with a high affinity for PS, binding to a Ca+-dependent phospholipid bilayer containing PS [70]. A total of 43% of apoptotic hepatocarcinoma cells (HepG2) after 48 h exposure to an ethyl acetate fraction from an ethanol extract (320 μg/mL) of the algae *Turbinaria conoides* was observed [71]. A total of 20% of apoptotic glioblastoma cells (A172) were detected after being exposed to a 250 μg/mL hexane extract from the algae *C. lentillifera* [72]. Arumugam et al. [73] observed 20–40% of apoptotic hepatocarcinoma cells (HepG2) using fucoidan at 50–200 μg/mL concentrations. A 96% ethanol extract of *C. racemosa* showed the induction of apoptosis in treated cells. It decreased HeLa cell viability at 24 h and 48 h post-treatment with a range of 50–200 μg/mL [74].

Regarding the cell cycle, it is essential to remember that DNA replication occurs in the S phase. In the G2 phase, the cell continues the biosynthetic metabolic phase, verifies the fidelity of the replicated DNA, and prepares to enter mitosis. Therefore, the arrest of the cell cycle in phase S indicates damage to the genetic material. CSE may be inhibiting that replication by interfering in its organization, inhibiting the regulatory proteins of this phase, or altering the signaling pathways. When the cell cycle stops during cell division, damage, and error are challenging to repair [75]. In addition, a marked cell arrest generated by CSE at 1000 μg/mL in phase G2/M after damage in phase S was observed; this arrest suggests that the remaining cells depended on the G2/M checkpoint to counteract and/or prevent the consequences of DNA damage occurring in phase S. All of this confirms the conclusions of Kuczler et al. [76] that the arrest of the cell cycle allows more extensive repairs of the DNA or apoptosis in the case of damages that extend beyond the point of repair. ROS is involved in cell cycle arrest by hindering the repair of damaged genetic material by inhibiting repair pathways [77]. The HPLC-MS analysis revealed the presence of different bioactive compounds that interfere with the cell cycle, for example, damnacanthal, an anthraquinone compound (alkaloid of the quinone family) with anticancer properties [78]. Li et al. [79] said damnacanthal induced cell cycle arrest by increasing p27 protein Kip1 levels in ovarian carcinoma cells SKVO3 and A2780. Kim et al. [80] showed that baicalein stopped the S-phase cell cycle of human colorectal cancer cells HCT-116. Likewise, baicalein caused cell cycle arrest in G1/S by inhibiting the Akt/mTOR pathway in H1299 and H1650 lung cancer cells, which decreased the expression of the proteins CDK2, CDK4, and cyclin E2 [81]. Likely, cell cycle arrest in the S and G2/M phases caused by CSE is due to the compounds damnacanthal and baicalein. To understand the mechanism of action of CSE on the cell cycle, the activity of the regulatory proteins of the S phases (cyclin E/CDK2) and G2 (Cyclin B/CDK1) and CDKIs, as well as the generation of ROS and the MAPK/AKT/mTOR signaling pathways, should be examined due to the essential role they play in the regulation of the cell cycle.

In carcinogenesis, a tumor microenvironment rich in extracellular ATP is created, consequently increasing the interaction of tumor cells and immune cells [39]. Chauvin et al. [82] showed that after 24 h exposure of human colon cancer spheroids to a plasma-activated medium, the ATP level was decreased by 70%. The ATP level of colon cancer cell spheroids (HT-29) decreased by 62 and 80% after 24 and 48h treatment exposure, respectively, when treated with 100 μg/mL of doxorubicin. Meanwhile, the antimicrobial peptide gramicidin decreased the ATP level only by 20 and 50% at 113 μg/mL concentration in the same period reported [83]. Likewise, Martínez-Rodríguez et al. [84] indicated a significant decrease in cell viability by ATP quantification after one, four, and seven days of naringenin exposure to different concentrations in spheroids of cervical cancer cells (HeLa).

Depolarization of the mitochondrial membrane induced by CSE is characteristic of a decrease in mitochondrial membrane potential. It should be noted that no studies of MMP performed in a three-dimensional model have been reported. Therefore, the possible comparisons are with monolayer culture data. A 62% loss of MMP from MCF-7 cells has been observed after 24 h exposure to a fucoidan extract [85]. Likewise, Ryu et al. [86] reported an increase in the depolarization of the mitochondrial membrane of colon cancer cells HCT116 using an 80% ethanol extract of the algae *Ulva fasciata*. Sakthivel et al. [87] reported a 58% decrease in MMP from lung adenocarcinoma cells, A549, after 24 h of phytol exposure. Similarly, a reduction in MMP using 100 μg/mL of ethanolic, methanolic, and hexane extracts of the algae *Enteromorpha compressa* on squamous cell carcinoma of the pharynx (FaDu) and squamous carcinoma of the tongue (Cal33) was observed [88]. Similar results have been reported using a 250 μg/mL hexane extract from the algae *C. lentillifera* on glioblastoma A172 cells [72]. Cancer cells consume more energy to survive and continue proliferating than cells under normal conditions. It is important to remember that this alteration has been identified as a hallmark of cancer [40]. Mitochondria is the energy provider for cancer cells, so it is considered one of the critical organelles in cancer therapy [89]. It should be emphasized that mitochondria play an essential role in apoptosis because it contains different proapoptotic molecules, such as cytochrome c, that can trigger the intrinsic pathway that leads to programmed cell death and molecules such as SMAC/DIABLO (second mitochondrial activator of caspases/direct IAP binding protein with low PI) that favor apoptosis by inhibiting IAPs (inhibitory apoptosis proteins) [33]. The loss of mitochondrial membrane potential is associated with the generation of reactive oxygen species (ROS) because the oxidative damage caused by ROS is probably a major cause of mitochondrial genomic instability and respiratory dysfunction [90]. Srinivas et al. [77] stated that elevated ROS levels could trigger apoptosis by generating ROS. As a result of oxidative stress, the pores along the mitochondrial membrane can be oxidized, or the mitochondrial membrane can be depolarized, causing the release of proapoptotic compounds into the cytoplasm and thus initiating the apoptotic program [77,91]. Therefore, it is likely that CSE might increase oxidative stress beyond the limit, thus triggering mitochondria-mediated apoptosis. Consequently, it was concluded that the mitochondrial damage observed is a result of CSE-induced apoptosis accompanied by the release of mitochondrial proapoptotic molecules into the cytoplasm and that the generation of ROS in cells can be examined to strengthen the results and better understand the mechanism of action of CSE.

Studying the caspases family is essential to understanding the mechanism and pathway of triggering the apoptotic program. An upward regulation of caspases-3, -8, and -9 in a 2D model of MCF-7 cells was reported after 24, 48, and 72 h exposure to a methanolic extract of *Sargassum muticum* [92]. Similar results were obtained by Gomes et al. [93] after having exposed a 2D model of HeLa cells to 500 μg/mL of methanol extract from the algae *Dictyota cilliolata* and *D. menstrualis*. Pradhan et al. [89] observed a positive regulation of caspases-3/7 using 100 μg/mL of ethanolic, methanolic, and hexane extracts of *Enteromorpha compressa* on squamous cell carcinoma of the pharynx (FaDu) and squamous carcinoma of the tongue (Cal33) from a 2D model. Martínez-Rodríguez et al. [84], did not record caspase activity -3/7, -8 and -9 after 12, 24 and 72 h of exposure of 136.13 μg/mL of naringenin on 3D model of HeLa cells. A 96% methanolic extract of *C. racemosa* at 200 μg/mL significantly increased the expression of pro-apoptotic proteins Bax and cleaved caspase-3 in a 2D culture model compared to the control [74]. The HPLC-MS showed different bioactive compounds with pro-apoptotic activity, of which baicalein can be mentioned. This flavone has excellent potential to treat and prevent cancer without causing severe side effects [94]. This compound activated caspases-3 and -9 in colon carcinoma cells, HT-29, and apoptosis [95]. Likewise, Klimaszewska-Wiśniewska et al. [96] reported that quercetin, a flavonol, induced apoptosis in lung adenocarcinoma cells A549 through the negative and positive regulation of the anti- and proapoptotic proteins Bcl-2 and Bax, respectively. Pinocembrin-induced apoptosis of A549 cells is accompanied by an upward increase in caspase-3 activity [97]. The pro (Bax, Bok, Bak, Bik, Blk, Bad, Bid, Puma, and Noxa) and antiapoptotic (Bcl-2, Bcl-XL, Bcl-w, Boo, and Mcl-1) proteins of the Bcl-2 family are known to regulate the mitochondrial pathway of apoptosis. The effect of CSE on caspase-9 is more evident than on caspase-8; the intrinsic pathway would preferably carry out the apoptotic process. The induction of apoptosis through the intrinsic pathway was correlated with the results obtained from the loss of MMP because this is an event before the activation of caspase-9. This activation occurs when the proapoptotic protein Bax is translocated to the mitochondrial membrane, inducing its permeability (MOMP: mitochondrial outer membrane permeabilization). In contrast, the antiapoptotic proteins maintain control of mitochondrial permeability by blocking the activity of the proapoptotic proteins of that family. MOMP allows the release of different molecular compounds, such as cytochrome c, to the cytosol that, together with dATP and Apaf-1, bind to procaspase-9 forming the apoptosome complex and converting procaspase-9 into its active form (caspase-9) [44]. Therefore, it is likely that intrinsic pathway-mediated apoptosis is due to the balance generated by quercetin between the Bax and Bcl-2 proteins accompanied by the effect of baicalein and pinocembrin on caspases-3 and -9. Therefore, the activity of pro- and anti-apoptotic proteins, including some essential protein compounds on the inner side of the mitochondrial membrane, can be examined to better understand CSE’s mechanism of action.

The greatest terror in carcinogenesis is the sequence of events that lead to metastasis. Metastasis is the spread of cancer cells from a primary tumor to secondary and tertiary tissues and/or organs. In addition, it is the main event that causes the death of most cancer patients [98]. Lee et al. [99] reported that 200 μg/mL of fucoidan extracted from the algae F. vesiculosus decreased the invasion of lung cancer cells A549 by 86% compared to the control in a 2D model after 48h of exposure. Similarly, a decrease in the invasion of colon carcinoma cells HT-29 in a 2D model by inhibiting MMP-2 after 48 h of exposure of these to 200 μg/mL of fucoidan was reported [100]. Martínez-Rodríguez et al. [84] reported that naringenin at 136.12 μg/mL significantly decreased the invasion of HeLa cancer cells in a three-dimensional model. It is important to remember that cancer cells express matrix metalloproteinases (MMPs), for example, MMP-2/-9, which degrade the ECM-generating pathways that allow migratory cells to invade freely [101]. These secrete substances that modify the expression of proteins that control motility and migration so that the tumor initiates the process of angiogenesis, without which it would not develop [47]. The HPLC-MS analysis revealed the presence of different bioactive compounds capable of inhibiting the invasive capacity of tumor cells. Li et al. [79] reported that damnacanthal inhibited migration and invasion in SKVO3 and A2780 ovarian carcinoma cells. According to Gao et al. [102], the proliferation and migration of ovarian cancer cells can be suppressed by pinocembrin through decreased expression of N-cadherin and the gamma-aminobutyric acid receptor, also confirming that treatment with pinocembrin led to a decrease in the proliferation, migration, and invasiveness of colorectal cancer cells. Barni et al. [65] reported that carnosic acid inhibited cell adhesion and migration of the colon cancer line Caco-2, possibly reducing the activity of secreted proteases such as urokinase plasminogen activators (uPAs) and metalloproteinases (MMPs). In addition, Klimaszewska-Wiśniewska et al. [96] confirmed that quercetin repressed the migration of A549 cells, proposing and explaining that the influence of quercetin disassembly on vimentin filaments, microtubules, and microfilaments accompanied by its suppressive effect on N-cadherin and vimentin expression could be responsible for reduced migration of A549 cells in response to quercetin therapy. Therefore, the decreased invasion in SKLU-1 cells might be due to these compounds’ activity on ECM-degrading proteins. Consequently, it is suggested to analyze the expression of essential proteins (MMP-2/9, N-cadherin, among others) that actively participate in the invasive and metastatic process to understand better how CSE directly inhibited the invasive potential of SKLU-1 cells.

## 4. Materials and Methods

### 4.1. Collection of Macroalgae

The specimen was collected at Carreyeros Beach, Bahía de Banderas, Nayarit, Mexico, in February 2022. The collected biological material was frozen in plastic bags in the Biopolymers laboratory of the Department of Biochemical Engineering of the National School of Biological Sciences-IPN Zacatenco Unit. The macroalgae were washed with distilled water to remove all epiphytes and impurities and stored in an ultra-freezer at −74 °C for further analysis. The observation of taxonomic characteristics and a histological examination of the specimen based on dichotomous keys in the literature were conducted to identify the species. The specimen’s internal and external morphology was analyzed using a microscope [103] for this identification.

### 4.2. Macroalgae Extract

Once the species was identified, *Caulerpa sertularioides* was freeze-dried for 72h at −50 °C and 0.014 mbar and sprayed to obtain a fine powder of average size less than 1/2 mm. Then, the extract was obtained using an ultrasonic bath for 1 h with cold stirring using 80% ethanol [104]. Finally, the extract was dried in centrifugal concentrators (Genevac TM miVac Duo Concentrator) for further trials.

### 4.3. Characterization of Macroalgae Extract

#### 4.3.1. Quantification of Total Phenolic Compounds

The content of total phenolic compounds was determined by the Folin-Ciocalteu method with some modifications. The researchers added 90 μL of 10% Folin-Ciocalteu reagent to 20 μL of *C. sertularioides* ethanol extract. After five min., 90 μL of Na_2_CO_3_ solution (60 g/L) was added to the mixture. Subsequently, the preparation was incubated for 90 min in a microplate reader (SYNERGY H1, BioTek, Winooski, VT, USA), and the absorbance was measured at 750 nm [105]. The content of phenolic compounds was expressed as mg of gallic acid (GAE) equivalent/g of sample.

#### 4.3.2. Quantification of Total Flavonoids

According to Fattahi et al. [106], the colorimetric method evaluated flavonoid content with some modifications. Briefly, 0.5 mL of *C. sertularioides* ethanol extract was mixed with 2 mL of distilled H_2_O and 150 μL of 5% NaNO_2_. After five min., 150 μL of 10% AlCl_3_ was added, and finally, 2 mL of NaOH at 0.5 M after three min. The mixture was then incubated for 30 min. The absorbance was read at 510 nm on a spectrophotometer (GENESYS 10S, Thermo Fisher Scientific, Waltham, MA, USA). The total flavonoid content was expressed in mg of quercetin (QE) equivalent/g of the sample.

#### 4.3.3. Quantification of Total Carotenoids

The total carotenoid content was evaluated by spectrophotometry according to some modifications by Osuna-Ruiz et al. [105]. Briefly, 0.01 g of powdered sample was mixed with 5 mL of acetone, leaving it to stand for 10 min. Then, 5 mL of petroleum ether was added to the mixture. The solution was washed with distilled water until the acetone was removed, and the wastewater was removed with anhydrous sulfate. Subsequently, absorbance was read at 450 nm using a spectrophotometer (GENESYS 10S, Thermo Fisher Scientific, Waltham, MA, USA). The total carotenoid content was expressed as μg/g of β carotene

X (μg/g) = A × y (mL) × 10^6^/A^1%1 cm^ × 100

x (μg/g) = x (μg)/sample weight (g) × FD

where:

A: Absorbance
y: Volume of the solution that gave the absorbance
A^1%1 cm^: Carotenoid absorption coefficient
FD: Dilution Factor

#### 4.3.4. Determination of Antioxidant Capacity by ORAC

With some modifications, oxygen radical absorption capacity (ORAC) was determined according to Quek et al. [107]. Briefly, 20 μL of a 10 mM fluorescein solution and 50 μL of 2,2-azobis (2-amidino-propane) dihydrochloride (AAPH) 12 mM were added to 20 μL of the *C. sertularioides* sample or 6-hydroxy-2,5,7,8-tetra-methylchroman-2-carboxylic acid (Trolox). The readings were performed by recording the loss of fluorescence every two minutes for two hours at an excitation length of 485 and 515 nm of emission using a microplate reader (SYNERGY H1, BioTek, Winooski, VT, USA). All samples were prepared in PBS at pH 7.4 in a 1:2000 ratio.

The ORAC value was calculated using the following equation, and the results were expressed as TEAC values (μmol of Trolox/g dry base sample).

ORAC value = [(AUC sample − AUC control)/(AUC Trolox − AUC control)] · FD

where:

AUC sample = area under the sample curve
AUC control = area under the control curve and
AUC Trolox = area under the curve using the Trolox as a standard sample
FD = dilution factor of extracts

#### 4.3.5. Liquid Chromatography Profiling Coupled to Mass Spectrometry (HPLC-MS)

It was carried out in agreement with Chia et al. [50] with some modifications. The CSE extract was subjected to HPLC-MS after testing its antitumor activity. The samples (500 μL) were injected and analyzed with the LCMS Triple Quadrupole system, Agilent brand model G6410 with dual ESI source. The solution was filtered with a 0.45 μm Nylon syringe filter. The dimension of the column used was 4.6 × 150 mm. The binary mobile phase consisted of solvents A (water with 0.1% formic acid) and B (100% acetonitrile). The flow rate was 10 L/min. The data were analyzed with Agilent MasHunter Qualitative Analysis B.05.00 software (https://www.agilent.com/en-us/support/software-informatics/masshunter-workstation-software/, accessed on 20 April 2023). The compounds were identified by searching METLIN: Metabolite and Tandem MS Database. The HPLC-MS parameters were as follows: ionization chamber temperature, 100 °C; gas temperature, 300 °C; capillary voltage, 4 KV; fragments voltage, 95 and 135 V; and gas flow, 10 L/min. The electrospray ionization (ESI) source was established in positive and negative modes to acquire all mass spectrometric data.

### 4.4. Antitumor Activity

#### 4.4.1. Cell Line

The SKLU-1 lung cancer cell line (ATCC^®^CCL-2™) was grown in RPMI GIBCO™ (Thermo Fisher Scientific) medium supplemented with 10% fetal bovine serum (FBS) GIBCO™ (Thermo Fisher Scientific) and 1% antifungal and antibiotic GIBCO™ (Thermo Fisher Scientific) under a temperature of 37 °C in an atmosphere of 5% CO_2_, with 95% of relative humidity.

#### 4.4.2. Study Design

**Figure 13:**
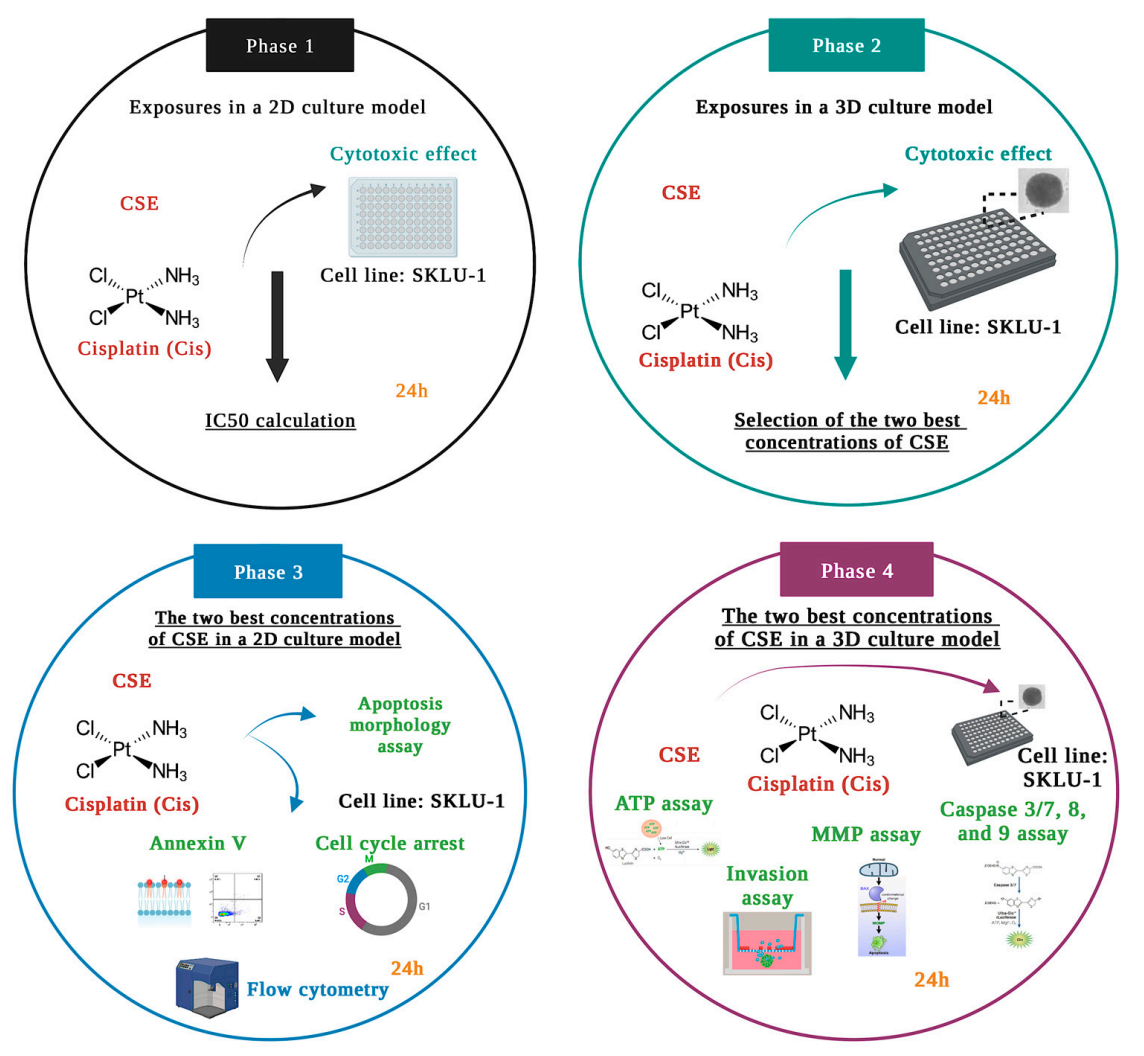
Schematic representation of the study design.

The experiments were carried out according to the study design divided into four phases, depicted in Figure 13.

In Phase 1, the cytotoxic effects of six CSE concentrations and one concentration of Cis were examined by the membrane permeability assay in the 2D culture model of lung adenocarcinoma cells SKLU-1. Considering the results obtained in Phase 1, (500, 800, and 1000 μg/mL, IC_50_x4, IC_50_x6, and IC_50_x12, respectively) were selected to be tested in a 3D culture model. According to the results obtained in Phase 2, the two best concentrations of CSE were chosen to conduct annexin V, apoptosis morphology, and cell-cycle studies in a 2D model. In Phase 4, these two concentrations were tested in 3D cultures by analyzing ATP level, mitochondrial membrane permeability, caspase activity, and cell invasion.

In all the experiments, control cells (negative control) were incubated in a culture medium with 0.1% DMSO.

#### 4.4.3. Cell Viability in a 2D and 3D Culture Model by Plasma Membrane Integrity

This trial was carried out according to Martínez-Rodríguez et al. [84] with some modifications. For the 2D model, 5.0 × 10^3^ cells were seeded in 96 wells of adhesion plates (Corning^®^). SKLU-1 cells were exposed to 25, 50, 75, 100, 150, 200 μg/mL CSE and 9.94 μg/mL of Cis for 24 h. After the treatment, the dye Sytox Green^®^ was added, and the fluorescence was read in a microplate reader (SYNERGY H1, BioTek, Winooski, VT, USA). The 3D model was carried out the same way as the 2D model with some modifications. The researchers seeded 1.2 × 10^3^ cells in ultra-low adhesion plates from 96 wells (Corning^®^). After the spheroid’s formation, the SKLU-1 cells were exposed to 500, 800, and 1000 μg/mL of CSE and 9.94 μg/mL of cisplatin for 24 h.

#### 4.4.4. Annexin V Test in a 2D Culture by Flow Cytometry

The annexin V test was performed with the Annexin V-FITC Apoptosis kit (Calbiochem^®^) following the manufacturer’s specifications. Briefly, 800 × 10^3^ cells per mL were seeded in 3.5 cm plates from 6 wells (Nest^®^) exposed to 800 and 1000 μg/mL of CSE and 9.94 μg/mL of cisplatin for 24 h. The cells were stained with annexin V-FITC and propidium iodide to be later analyzed in BD LSRFortessa cytometer (BD Biosciences).

#### 4.4.5. Nuclear Staining with Hoechst 33258 for the Study of Cell Morphology

This trial was performed as described by Wang et al. [95] with some modifications. Briefly, 5 × 10^3^ cells were seeded in 96-well (Corning^®^) plates exposed to 800, 1000 μg/mL of CSE, and 9.94 μg/mL of cisplatin for 24 h. Subsequently, the harvested cells were washed with PBS and fixed with 4% paraformaldehyde for 20 min. The cells were stained with Hoechst 33258 for 10 min, then observed under an inverted microscope Zeiss Axiovert 25 (Carl Zeiss) at a magnification of 20× and analyzed in the ImageJ software (http://imagej.nih.gov/ij, accessed on 20 April 2023).

#### 4.4.6. Cell Cycle Test in a 2D Model by Flow Cytometry

This trial was performed according to Gomes et al. [93] with some modifications. The SKLU-1 cells were seeded in a 3.5 cm plate of 6 wells (Nest^®^) (200 × 10^3^/mL). After 24 h of exposure to the treatments, these were harvested and fixed in 4% paraformaldehyde for 60 min. Subsequently, Rnase A and propidium iodide (30 μg/mL) was added. The DNA content was analyzed using a BD LSRFortessaTM (BD Biosciences) flow cytometer. A total of 20,000 events were purchased. For data analysis, FlowlogicTM Analysis Software, version 8.6, was used. The data presented are representative of those obtained in three independent experiments conducted in duplicate.

#### 4.4.7. ATP Quantification in a 3D Culture Model

The cell viability assay by ATP quantification was performed with the CellTiter-Glo^®^ kit, Reagent 2.0 (Promega Corp., Madison, WI, USA), following the manufacturer’s specifications. Briefly, 300 cells were seeded per well. After 24 h of exposure to the spheroids with 800 and 1000 μg/mL of CSE and 9.94 μg/mL of cisplatin, the CellTiter-Glo^®^ reagent was added and incubated for 30 min. to then quantify the luminescence in a microplate reader (SYNERGY H1, BioTek). The detection is based on using the luciferin-luciferase reaction to measure the amount of ATP of viable cells where the amount of light detected is directly proportional to the ATP content and indicates the presence of metabolically active (viable) cells.

#### 4.4.8. Mitochondrial Membrane Potential (ΔΨm) Assay in a 3D Model

This trial was conducted in agreement with Wang et al. [95] with some modifications. The researchers seeded 1.2 × 10^3^ cells in ultra-low adhesion plates from 96 wells (Corning^®^). After forming the spheroid, it was exposed to 800 and 1000 μg/mL of CSE and 9.94 μg/mL of cisplatin for 24 h. Subsequently, rhodamine 123 was added, and fluorescence was read in a microplate reader (SYNERGY H1, BioTek).

#### 4.4.9. Caspases 3/7, -8, and -9 Test in a 3D Model

Caspase activity was determined with the Caspase-Glo^®^, -3/7, -8, and -9 kit (Promega Corp., Madison, WI, USA) following the manufacturer’s specifications. Briefly, 300 cells were seeded per well, and after 24 h of exposure to the spheroids with 800 and 1000 μg/mL of CSE and 9.94 μg/mL of cisplatin, the corresponding Caspase-Glo^®^ reagent was added. Subsequently, it was incubated for an hour and quantified the luminescence in a microplate reader (SYNERGY H1, BioTek). The luminescence detected is proportional to the amount of caspase activity present.

#### 4.4.10. Invasion Test in a 3D Culture Model

The invasion test was performed as described by Martínez-Rodríguez et al. [84] with some modifications. Briefly, 1.2 × 10^3^ cells were seeded per well, and after the formation of the spheroid, it was treated with 800 and 1000 μg/mL of CSE and 9.94 μg/mL of cisplatin for 24 h. Subsequently, 100 μL of Matrigel^®^ Matrix (Corning^®^) per well and then RPMI medium was added. The invasion was determined using an inverted Zeiss Axiovert 25 microscope (Carl Zeiss) and ImageJ software and calculated by measuring the area and perimeter between the spheroids’ edge and the invading cells’ edge.

## 5. Conclusions

The results found in this work show that *C. sertularioides* extract is a potential therapeutic adjuvant capable of significantly reducing cell viability in SKLU-1 lung cancer spheroids. In addition, it generated mitochondrial damage and induced apoptosis through both signaling pathways. CSE also caused biochemical and morphological changes typical of apoptosis and directly inhibited the invasive potential of SKLU-1 tumor cells. It stopped the cell cycle in the S and G2/M phases in response to genetic damage by preventing damaged DNA from replicating further and thus inhibiting the proliferative capacity of cells. As observed, CSE has antioxidant, antiproliferative, proapoptotic, and anti-invasive abilities, so it becomes a potential candidate for an alternative treatment with bioactive compounds that can act as adjuvants, decreasing the dose of chemotherapeutic drugs and, at the same time, the aggressivity of them. Additional studies are required to strengthen and better understand CSE’s mechanism of action on lung cancer’s carcinogenesis.

## 6. Recommendations for Future Work

The current study suggests using the CSE’s primary component, caulerpin, to determine its effect and mechanism of action. A multi-approach with molecular and histological analysis and combination with first-line drugs must be included.

## Figures and Tables

**Table 1:** The total phenols, flavonoids, carotenoids, and antioxidant capacity were obtained from *C. sertularioides* 80% ethanol extract (CSE).

Total Phenol (mg Eq. GAE/g)	Total Flavonoids (mg Eq. QE/g)	Total Carotenoids (μg Eq. β Carotene/g)	Antioxidant Capacity per ORAC (μmol TE/g)
81.09 ± 2.28	70.11 ± 2.06	207.56 ± 2.67	2171.21 ± 1.35

Characterization of CSE ethanol extract.

**Table 2:** CSE compounds were identified from a negative ionization mode.

Compound	Compound Class	RT	M-1	M
Caulerpin	Alkaloid	17.7	397	398
Difucol	Phlorotannin	20.6	249	250
Quercetin	Flavonol	22.1	301	302
Dihydroquercetin	Flavonol	23.6	303	304
P-coumaroyl malic acid	Phenolic compound	25.4	279	280
Gallocatechin	Flavonol	26.4	305	306
Carnosic acid	Phenolic diterpene	27.6	331	332
Stigmasterol ferulatol	Sterol	28.1	591	592
Damnacanthal	Alkaloid	30.1	281	282
Pinocembrin	Dihydroxyflavanone	30.5	255	356
Baicalein	Flavone	34.1	445	446

Compounds detected in a negative ionization mode. RT: Retention Time.

**Table 3:** CSE compounds were identified from a positive ionization mode.

Compound	Compound Class	RT	M + 1	M
Caulerpin	Alkaloid	17.7	399	398
Difucol	Phlorotannin	21.5	611	610
Quercetin	Flavonol	22.1	593	592

Compounds detected in a positive ionization mode. RT: Retention time.

**Table 4:** Cell cycle distribution of SKLU-1 cells treated with CSE and cisplatin.

Treatments	G0/G1	S	G2/M
Control	55.59 ± 0.45	35.57 ± 0.12	8.86 ± 0.41
Starvation	53.37 ± 0.53	32.55 ± 0.37	14.08 ± 0.78
CSE [800 µg/mL]	10.08 ± 0.19	67.59 ± 0.18	22.34 ± 0.38
CSE [1000 µg/mL]	1.38 ± 0.06	88.20 ± 0.21	10.42 ± 0.19
Cisplatin [9.94 µg/mL]	1.41 ± 0.08	92.72 ± 0.34	5.87 ± 0.31

S and G2/M phase cell cycle arrest by CSE in a 2D model culture of SKLU-1 lung cancer. SKLU-1 cells were exposed to 800, 1000 μg/mL CSE, and 9.94 μg/mL of cisplatin for 24 h. Data represent mean values and standard deviation from independent experiments (*p* ≤ 0.05) *n* = 3.

**Table 5:** Cytotoxic effect of caulerpin isolated from various algae genera on different cancer cell lines in a 2D culture model.

Chemical Structure	Sources	Cell Lines	Type of Cell Lines	IC50 [μg/mL]	Treatment Time	References
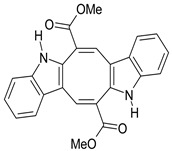	*Caulerpa peltata*	L5178Y	mouse lymphoma cells	12.2	72 h	[59]
	Purchased from Yuanye Pharmaceutics (Shanghai, China)	LOVO	Colon Cancer	7.97	48 h	[60]
		SW480		12.35		
	*Sargassum platycarpum*	HepG2	Hepatocarcinoma	24.6	24 h	[61]
	*Halimeda cylindracea*	NCL-H460	Lung Cancer	20.05	48 h	[62]
	*Caulerpa cylindracea*	HCT-116	Colon Cancer	47.4	48 h	[63]
		HT-29		71.34		

Effect of caulerpin isolated and purified from different seaweed species on various cancer cell lines.

**Table 6:** Comparative IC_50_ [μg/mL] table in 2D and 3D models of different lung carcinoma cell lines.

	IC_50_ Value [μg/mL]								
Drugs/Cell Lines	H460 Cells		A549 Cells		H1650 Parental Cells		H1650 Stem Cells		Authors
2D/3D Models	2D	3D	2D	3D	2D	3D	2D	3D	
Camptothecin	0.90 ± 0.26	24.29 ± 2.72	0.47 ± 0.06	31.26 ± 2.59	1.56 ± 0.28	18.06 ± 1.68	2.61 ± 0.36	33.25 ± 3.72	[66]
Cisplatin	1.04 ± 0.13	25.37 ± 1.69	1.26 ± 0.06	22.82 ± 1.36	0.63 ± 0.29	19.91 ± 2.23	1.46 ± 0.19	37.98 ± 3.74	
Doxorubicin	0.76 ± 0.15	41.45 ± 4.63	1.05 ± 0.19	64.19 ± 6.75	1.47 ± 0.36	44.54 ± 3.44	7.90 ± 0.67	82.09 ± 8.55	
Gemcitabine	0.61 ± 0.04	23.97 ± 1.84	0.67 ± 0.12	22.98 ± 2.54	0.70 ± 0.15	27.30 ± 2.55	1.59 ± 0.22	46.79 ± 3.69	
5-Fluorouracil	0.47 ± 0.06	9.07 ± 1.02	0.18 ± 0.02	11.67 ± 0.97	0.58 ± 0.10	6.74 ± 0.62	0.97 ± 0.14	12.42 ± 1.39	
**CSE on SKLU-1 cell line**									
**2D model**				**3D model**					
IC_50_ = 80.28 μg/mL				IC_50_ = 530 μg/mL					

Comparative table of IC50 in 2D and 3D models.

## Data Availability

Data are available upon request to the corresponding author.

## Sample Availability

Samples of the compounds are available from the authors.
